# Photoacoustic Imaging in Immune-Mediated Inflammatory Skin Diseases: Diagnostic and Therapeutic Applications

**DOI:** 10.3390/diagnostics16172716

**Published:** 2026-08-25

**Authors:** Nafeixia Maimaitiyili, Xiaochun Lin, Jianping Wei, Xinyi Li, Jinyi Deng, Qiuting Zheng

**Affiliations:** 1 Non-Invasive Skin Imaging Center, Shenzhen Center for Chronic Disease Control, Shenzhen Dermatology Hospital, Shenzhen Institute of Dermatology, Shenzhen 518020, China; 2251482@tongji.edu.cn (N.M.); klaylin@163.com (X.L.); 13516534250@163.com (J.W.); 2250733@tongji.edu.cn (X.L.); 2250573@tongji.edu.cn (J.D.); 2 Department of Medical Ultrasound, Shanghai Skin Disease Hospital, School of Medicine, Tongji University, Shanghai 200443, China

**Keywords:** photoacoustic imaging, immune-mediated skin diseases, psoriasis, atopic dermatitis, treatment monitoring

## Abstract

**Background/Objectives**: Immune-mediated inflammatory skin diseases (IMSDs), including psoriasis, atopic dermatitis, and systemic sclerosis, are chronic relapsing disorders that impose substantial physical, psychological, and economic burdens. Current assessment relies largely on subjective clinical scoring and conventional imaging modalities that provide limited functional information. This structured narrative review evaluates photoacoustic imaging (PAI) as a non-invasive optical-ultrasound technique for morphological and functional assessment in IMSDs. **Methods**: We searched PubMed studies published from January 2017 to December 2025 using terms related to photoacoustic imaging, optoacoustic mesoscopy, psoriasis, atopic dermatitis, systemic sclerosis, contact dermatitis, and immune-mediated inflammatory skin disease. Studies were narratively synthesized by disease, PAI modality, comparator, outcome, and translational limitation. **Main Findings**: PAI can map vascular and pigment-related optical absorption and estimate oxygenation, blood volume, and selected structural biomarkers. In psoriasis, atopic dermatitis, and systemic sclerosis, PAI-derived measures have been associated with clinical severity, subclinical vascular or epidermal changes, and treatment response. However, most available studies are small, single-center, heterogeneous, or based on prototype systems, and several biomarkers still require histopathological and external validation. **Conclusions**: Current evidence supports PAI as a feasible research and potential adjunctive assessment tool for IMSDs rather than an established replacement for standard clinical, histopathological, or imaging methods. Standardized protocols, validation across skin phototypes, cost-effective devices, and prospective multicenter studies are required before routine clinical adoption.

## 1. Introduction

The skin serves as a physical barrier for the body and functions as an active immune organ. Keratinocytes, resident immune cells, blood vessels, and nerves sense injury, infection, and environmental stimuli, and coordinate local inflammatory responses. Sustained or dysregulated protective responses trigger cytokine- and chemokine-mediated inflammation, which disrupts the cutaneous microenvironment and drives the pathogenesis of immune-mediated inflammatory skin diseases (IMSDs) [1,2].

IMSDs are cutaneous disorders driven by genetic predisposition, environmental triggers, immune-cell activation and tissue remodeling, and are characterized by chronicity, recurrence and systemic involvement, including psoriasis, atopic dermatitis (AD) and systemic sclerosis (SSc). These conditions impose persistent itch, pain, sleep disturbance, work loss and impaired quality of life, and moderate-to-severe disease is frequently accompanied by systemic comorbidities and psychological distress such as depression and anxiety [3,4,5].

Given the substantial burden IMSDs impose on patients and society, effective management is imperative. Current therapeutic options primarily encompass pharmacological agents, such as glucocorticoids, calcineurin inhibitors, and systemic immunosuppressants, alongside ultraviolet phototherapy. In recent years, research into shared immunopathogenic mechanisms (e.g., T cell-mediated inflammation, cytokine dysregulation, and JAK signaling pathways) has accelerated the development of targeted biologics and small-molecule drugs, gradually shifting the therapeutic paradigm toward precision medicine [6]. However, the high cost of therapy and low drug survival due to inadequate response (which varies by age, family history, sex, and other patient-specific factors) constrain the application of biologics. These limitations underscore the need for precise, longitudinal assessment tools to enable early intervention, real-time efficacy monitoring, and long-term disease management.

**Figure 1:**
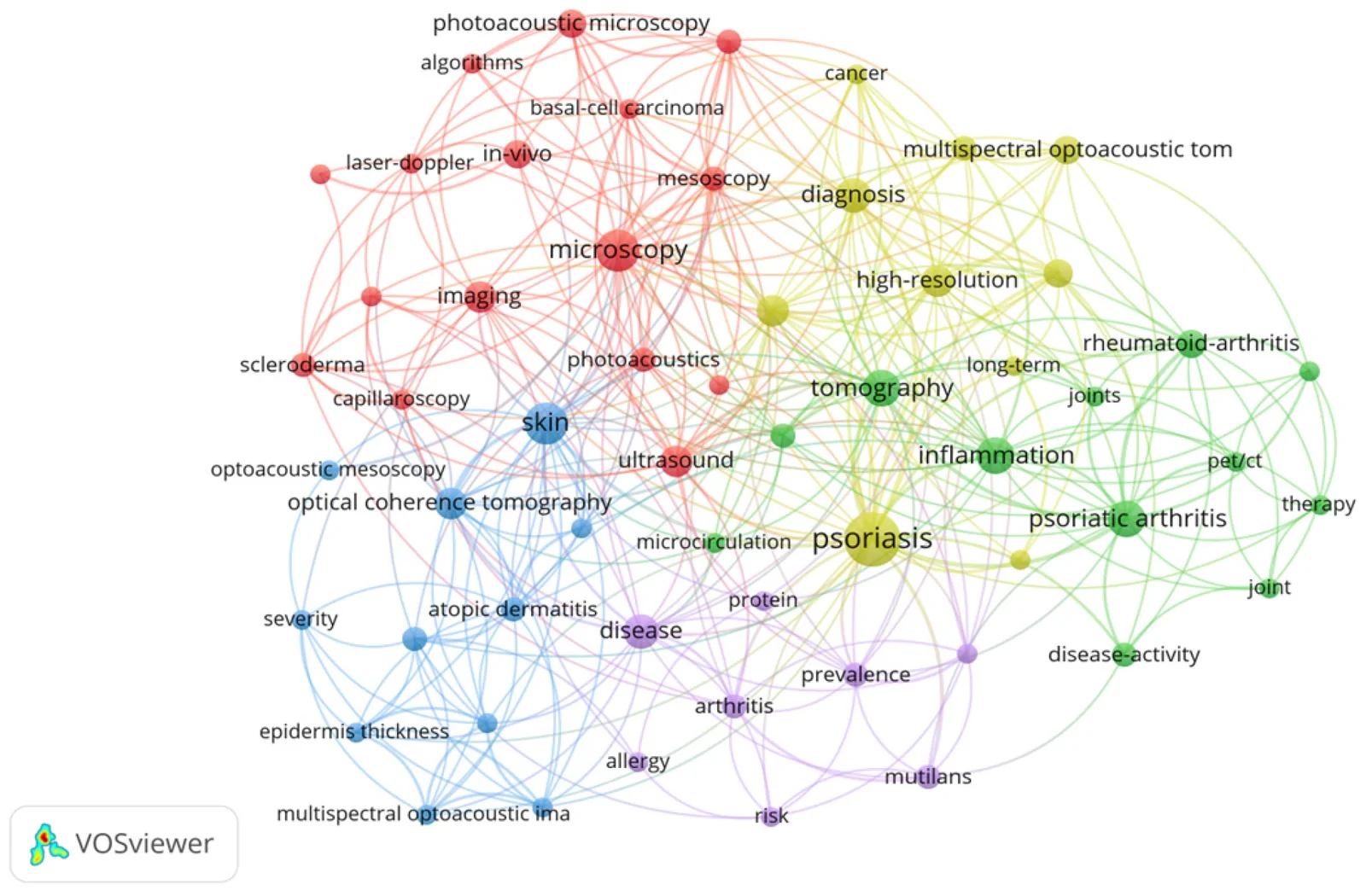
Keyword co-occurrence map generated with VOSviewer (1.6.18) from the included literature. Each node represents a keyword; node size reflects frequency; link thickness reflects co-occurrence strength; colors represent clusters.

**Figure 2:**
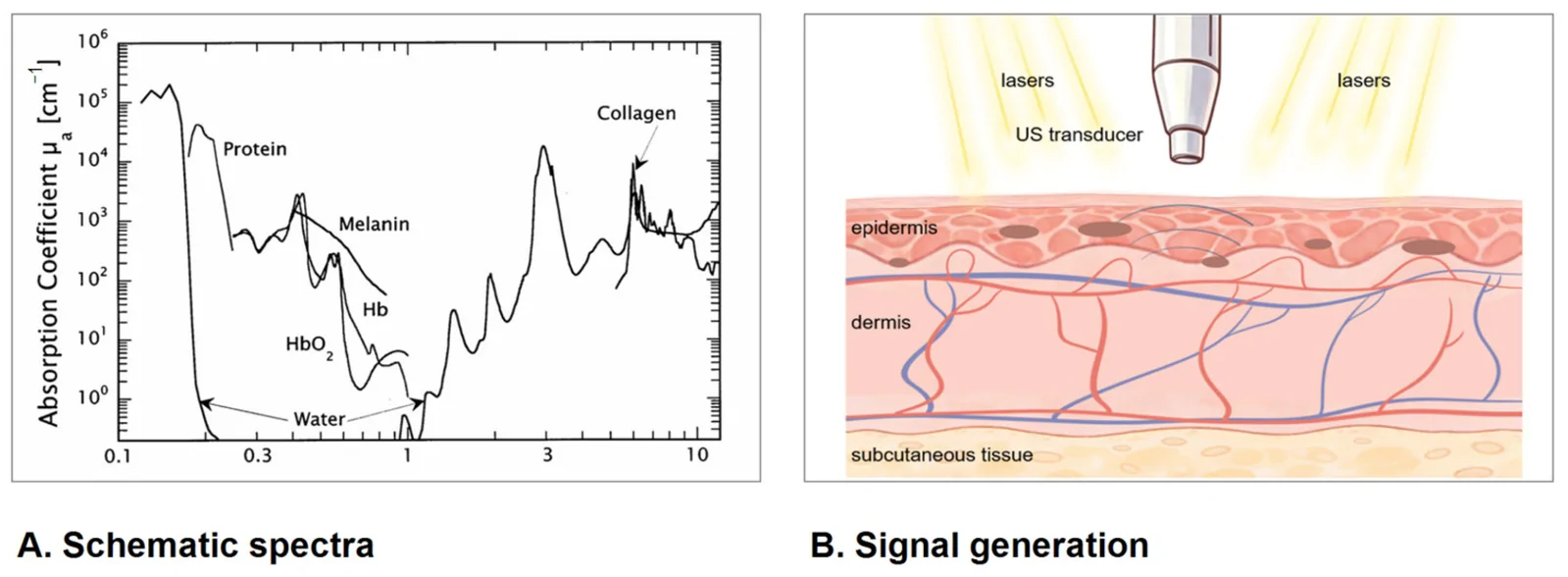
Photoacoustic contrast and signal generation in skin. (**A**) Schematic spectra of endogenous skin chromophores (reproduced from Ref. [9]). (**B**) Signal generation. Wavelength-selectable pulsed laser excitation is absorbed by endogenous chromophores and generates ultrasound that is detected by a transducer.

To provide an overview of the literature landscape, a keyword co-occurrence map of the studies included in this review is shown in Figure 1. As an emerging non-invasive imaging modality, PAI has been studied in the diagnosis and research of various diseases, including breast tumors, melanoma, inflammatory bowel disease, lymphedema, and arthritis [7,8]. It is based on the thermoelastic effect: when short-pulsed laser irradiation is delivered to tissue, endogenous chromophores such as hemoglobin and melanin absorb optical energy, undergo transient thermoelastic expansion, and generate ultrasonic waves detected by an ultrasound transducer (Figure 2B) [9]. The main skin chromophores relevant to PAI include hemoglobin, melanin, collagen and lipids; their wavelength-dependent absorption spectra are summarized in Figure 2A. By acquiring signals at selected wavelengths and applying post-processing methods such as spectral unmixing, PAI can visualize the spatial distribution of these absorbers and provide morphological and functional information.

Accordingly, this narrative review aims to synthesize disease-specific evidence on PAI in immune-mediated inflammatory skin diseases, clarify which biomarkers are supported by current data, and identify methodological and translational barriers that must be addressed before clinical adoption.

## 2. Materials and Methods

### 2.1. Search Strategy and Review Period

We used PubMed to search for studies evaluating PAI in IMSDs, published from January 2017 to December 2025. The search combined technology-related terms—“photoacoustic imaging”, “optoacoustic imaging”, “raster-scan optoacoustic mesoscopy”, “photoacoustic tomography”, and “photoacoustic microscopy”—with disease-related terms: “psoriasis”, “atopic dermatitis”, “systemic sclerosis”, “dermatitis”, and “immune-mediated inflammatory skin disease”. Terms within each category were connected by OR, and categories were linked by AND. Reference lists of included articles were also hand-searched for additional relevant studies.

### 2.2. Eligibility Criteria

Eligible articles included clinical studies, preclinical investigations, technical reports with dermatologic relevance, and reviews that informed the interpretation or translation of PAI in IMSDs. These reviews were not subject to primary data extraction but were used to contextualize findings. We prioritized studies reporting disease-related structural or functional biomarkers, treatment monitoring, comparison with established assessment methods, or technical limitations. Records were excluded if they did not involve skin or skin-relevant inflammatory disease, did not use PAI/optoacoustic technology, were unrelated conference abstracts without usable methodological detail, or lacked direct relevance to the review question.

### 2.3. Study Selection and Data Extraction

Articles were screened by title/abstract and then by full text. Extracted items included disease indication, sample size or model, PAI modality, comparator, principal biomarkers, main findings, and stated limitations. Because the field remains heterogeneous and many studies are feasibility-oriented, results were synthesized narratively rather than pooled quantitatively.

### 2.4. Assessment of Review Quality, Study Quality and Risk of Bias

The review followed the principles of a structured narrative review, with attention to the SANRA domains of justification, search description, referencing, scientific reasoning and appropriate presentation of data. Primary studies were appraised qualitatively using relevant domains from JBI, NOS or CASP tools, depending on study design. Particular attention was paid to sample size, patient selection, comparator choice, external validation, skin phototype representation, device standardization and reproducibility.

### 2.5. Brief Overview of Photoacoustic Imaging Modalities

As illustrated in Figure 2B, all PAI systems generate ultrasound from pulsed optical absorption, but they differ in illumination, acoustic detection and scanning geometry. Photoacoustic tomography (PAT) is generally used for deeper imaging with lower spatial resolution, whereas photoacoustic microscopy (PAM) and photoacoustic mesoscopy (PAMes) are more suitable for superficial skin structures. Raster-scan optoacoustic mesoscopy (RSOM), a form of PAMes, uses broadband high-frequency detection to resolve capillaries and larger vessels across the epidermis, dermis and superficial subcutis; however, it has a smaller field of view and slower scanning than some tomographic systems. These modality-dependent differences explain why the studies summarized in Table 1 used PAT, PAM, PAMes, RSOM or hybrid PA/HFUS systems for different disease questions.

## 3. Results

### 3.1. Diagnostic Applications

#### 3.1.1. Psoriasis

Psoriasis is a chronic systemic inflammatory disorder primarily characterized by localized or widespread scaly erythematous lesions on the skin. Current evaluation methods rely primarily on visual observation of skin lesions and lack quantitative analysis of cutaneous-layer morphology and vascular alterations. To contextualize the role of PAI, Table 2 compares conventional cutaneous imaging modalities with PAI for skin assessment. PAI can clearly visualize features such as epidermal spinous layer thickening, marked elongation and widening of dermal capillary loops, and dilation of superficial vascular plexuses in patients with psoriasis. Quantitative biological parameters can be extracted from lesion images, including epidermal thickness, total dermal blood volume, capillary loop density, mean diameter, and blood oxygen saturation; these parameters differ significantly between healthy individuals and patients with psoriasis [10,11,12]. Aguirre et al. proposed the optoacoustic index (OPIND) based on these parameters, which showed a strong correlation with the Psoriasis Area and Severity Index (PASI). Furthermore, PAI can sensitively reflect the progression of inflammation within the cutaneous layers of patients with psoriasis. Li et al. found that skin oxygen saturation measured by PAI was positively correlated with psoriasis severity. Even in non-lesional skin from severely affected patients, elevated oxygen saturation and total blood volume were observed, suggesting that PAI can identify subclinical lesions before overt cutaneous morphological changes emerge, thereby facilitating early disease assessment. However, as this study relied primarily on clinical diagnosis without histopathological confirmation, its conclusions require further validation.

Psoriasis can involve the joints, with diagnosis based on clinical musculoskeletal inflammatory symptoms combined with the Classification Criteria for Psoriatic Arthritis (CASPAR) [20]. The primary radiographic indicator in the CASPAR criteria is abnormal ossification; however, local anatomical destruction and pathological ossification often occur later than metabolic changes in joint tissue, and irreversible joint damage may already have occurred by the time these structural abnormalities are detected. As a functional imaging modality, PAI can identify patients with psoriatic arthritis (PsA) earlier than conventional X-ray examinations [13]. A cross-sectional study demonstrated that, compared with healthy controls, patients with psoriasis without joint involvement exhibited elevated levels of oxygenated hemoglobin and blood oxygen saturation at tendon insertion sites. These markers were even more markedly elevated in patients with PsA. The researchers suggested that this pattern reflects high perfusion and low metabolism in local tissues, which may further drive bone destruction and explain why metabolic changes precede structural bone alterations. Furthermore, PAI observations revealed that local tenderness and ossification correspond to reduced collagen content and increased lipid content, respectively. This finding is consistent with metabolic remodeling processes in arthritis, including neovascularization, collagen degradation, and adipose tissue replacement, as confirmed by previous laboratory studies [14].

#### 3.1.2. Atopic Dermatitis

Atopic dermatitis (AD) is a chronic inflammatory skin disorder and one of the most common subtypes of eczema. It is primarily associated with impaired skin barrier function and dysregulated immune responses. Currently, clinical assessments of AD severity rely predominantly on scoring systems, such as the Eczema Area and Severity Index (EASI) and the Scoring of Atopic Dermatitis (SCORAD). However, these subjective scoring methods depend heavily on the examiner’s clinical experience and are prone to inter- and intra-observer variability, limiting their accuracy and reproducibility [21].

PAI offers a more objective, quantitative approach for evaluating dermal vascular status and epidermal barrier function, with excellent accuracy and reproducibility [15,22]. Specifically, parameters such as blood oxygen saturation and total blood volume are closely correlated with the degree of skin inflammation, while epidermal thickness reflects the integrity of the epidermal barrier. Studies have shown that these PAI-derived parameters progressively increase as AD severity worsens. In patients with severe AD, the epidermal thickness of non-lesional skin approaches that of lesional areas—suggesting that subclinical epidermal barrier dysfunction may already be present in these seemingly unaffected regions. This finding provides valuable guidance for clinical treatment and underscores the critical role of skin barrier disruption in the pathogenesis of AD.

Furthermore, PAI-derived quantitative metrics are highly compatible with computer-assisted technologies. Previous studies have leveraged PAI parameters to train artificial intelligence (AI) models, which can assist in the objective assessment of AD severity [16,23]. Collectively, PAI provides robust, objective quantitative indicators for evaluating skin inflammation and epidermal barrier impairment in AD patients, while also facilitating integration with AI technologies to further optimize disease severity assessment.

#### 3.1.3. Systemic Sclerosis

Systemic sclerosis (SSc) is an autoimmune disorder that affects multiple organs, including the skin, and is primarily characterized by inflammation, vascular lesions, and fibrosis. The main cutaneous manifestations include Raynaud’s phenomenon, skin sclerosis, telangiectasia, and digital ulcers. Vascular alterations precede the development of fibrosis; therefore, nailfold capillaroscopy serves as a crucial tool for early diagnosis and prognostic assessment. In the early stages of SSc, nailfold capillaroscopy reveals dilated and tortuous capillaries. In contrast, in the advanced stages, as fibrosis progresses, the number of capillaries gradually decreases. However, the diagnostic performance of nailfold capillaroscopy may be compromised in patients with a thick stratum corneum or darker skin pigmentation. In such cases, PAI can serve as an important complementary imaging modality. It can provide comprehensive visualization of the microvascular structure of the nailfold region and, by quantifying vascular volume, vascular density, and capillary diameter, provide objective evidence for disease assessment [18,24].

Raynaud’s phenomenon is frequently the initial clinical manifestation of SSc; however, it is not disease-specific and can occur in primary Raynaud’s phenomenon and other connective tissue disorders. Therefore, clinical diagnosis requires differentiation from primary Raynaud’s phenomenon and other related conditions. Studies have demonstrated that blood oxygen saturation in patients with early-stage SSc is significantly lower than that in those with primary Raynaud’s phenomenon, indicating that PAI holds great potential for early differential diagnosis [19]. Compared with healthy individuals, patients with SSc generally exhibit significantly lower levels of oxygenated hemoglobin and blood oxygen saturation in the fingers, along with significantly greater skin thickness and mean elastic modulus [25,26]. Furthermore, dynamic testing has revealed that changes in these cutaneous biomarkers can reflect impaired arterial function in patients with SSc.

Eisenbrey et al. utilized PAI to record fingertip oxygenation changes in SSc patients and healthy controls before cold stimulation, as well as at 5, 15, and 30 min post-stimulation. They observed that fingertip oxygenation levels in SSc patients decreased significantly at all three time points following cold exposure [27]. In another study, Wilkinson et al. simulated digital vascular occlusion and found that SSc patients exhibited poorer fingertip oxygenation recovery after the occlusion was removed [28].

#### 3.1.4. Other Diseases

Sujino et al. [29] conducted a preliminary study using three-dimensional (3D) photoacoustic imaging on multiple cases of IMSDs. The findings showed that vitiligo is primarily characterized by reduced melanin signal, although increased hemoglobin signal in the superficial dermis may be observed in some cases. In contrast, purpura is marked by a decrease in cutaneous hemoglobin signal, which may be associated with vascular wall rupture. Reticular purpura in nodular polyarteritis is characterized by increased and remodeled dermal blood vessels. Notably, for localized skin lesions such as reticular purpura, traditional histopathological biopsies only reflect localized thickening of vascular walls and inflammatory infiltration. In contrast, 3D photoacoustic imaging can non-invasively visualize the overall architecture of subcutaneous microcirculation, thereby deepening our understanding of the pathophysiological mechanisms underlying this disease.

Patch testing is widely used for the diagnosis of contact dermatitis. Clinicians typically interpret test results through visual and tactile examination; however, this approach is subjective. Both irritant and allergic reactions can yield positive results, making it challenging to distinguish between the two conditions. Hindelang et al. [30] were the first to use RSOM to interpret patch test results with the aim of improving diagnostic accuracy. Their findings indicated that while there was no significant difference in cutaneous blood volume per unit area between the two types of reactions, allergic reactions were associated with more pronounced capillary dilation and tortuosity in the dermal papillary layer. This was specifically reflected by a decreased ratio of low-frequency to high-frequency signals and an increase in vascular fragments. A discriminant model developed based on these two parameters can assist in differentiating between irritant and allergic reactions.

### 3.2. Therapeutic Applications

#### 3.2.1. Treatment Monitoring

Photoacoustic imaging serves not only as a tool for assessing disease status but also as a reliable method for treatment monitoring; changes in its associated indicators provide a robust basis for evaluating therapeutic efficacy. During the clinical management of patients with atopic dermatitis and psoriasis, PAI showed that treated patients exhibited reduced epidermal thickness, decreased cutaneous blood volume, and smaller capillary diameters. Notably, even when no significant changes were observed in the clinical indicators of some patients, photoacoustic data could still capture improvements in subcutaneous vascular structure [11,17,31]. Furthermore, Ishida et al. [31] demonstrated that improvements in venous oxygenation following treatment may precede changes in vascular morphology. These biomarker changes provide timely feedback on treatment progress, thereby supporting evidence-based clinical decision-making.

A variety of emerging therapeutic approaches are currently being introduced. In this context, precise treatment-monitoring methods are particularly critical because they help clinicians compare the efficacy of different treatment regimens more effectively. In a comparative study of conventional versus biologic therapies for psoriasis, both treatment groups demonstrated therapeutic effects; however, the biologic treatment group showed a more significant reduction in PAI-derived indices, which objectively confirmed the efficacy of biologic agents [11].

#### 3.2.2. Treatment Dose Planning

Ultraviolet (UV) irradiation therapy can induce T-cell apoptosis and suppress pro-inflammatory cytokines, making it widely used in the clinical management of inflammatory skin diseases such as psoriasis and atopic dermatitis [32]. Clinicians typically determine phototherapy doses based on the minimal erythema dose (MED), defined as the lowest UV dose that produces perceptible erythema at the irradiated site 24 h after exposure. However, this conventional approach relies primarily on visual observation, which is prone to inter-observer variability and lacks objective quantitative support [33].

#### 3.2.3. Evaluation of Topical Anti-Inflammatory Agents

Conventional topical medications, represented by corticosteroids, primarily exert anti-inflammatory and anti-proliferative effects by constricting blood vessels and suppressing proliferative responses; therefore, PAI can visualize the pharmacokinetic characteristics of these agents in vivo. Kim and colleagues conducted and in vivo experimental studies of topical corticosteroids: after applying corticosteroids to mouse skin, they used photoacoustic microscopy (PAM) to monitor dynamic changes in the skin microcirculation. During the study, they observed that corticosteroids primarily induce constriction of cutaneous capillaries and small blood vessels, with the depth of drug action varying significantly depending on the administration route—subcutaneous injection mainly acts on the reticular layer of the dermis, whereas topical application primarily targets both the papillary and reticular layers of the dermis [34].

In a separate experiment, the research team evaluated the onset time and therapeutic efficacy of four common topical glucocorticoids (0.05% clobetasol propionate ointment, 0.1% mometasone furoate ointment, 0.1% triamcinolone acetonide ointment, and 1% hydrocortisone solution) using PAI-derived parameters. The results were consistent with those of previous clinical studies, confirming the reliability of PAI in assessing drug efficacy [35]. Compared with traditional evaluation methods that rely on subjective observation of skin blanching (a surrogate marker of vasoconstriction), PAI can directly visualize the therapeutic effects of topical anti-inflammatory agents, quantify vascular constriction, and provide objective quantitative evidence for precise clinical evaluation of drug efficacy.

## 4. Discussion

### 4.1. PAI in IMSDs

#### 4.1.1. Correlations Between PAI Signals and IMSDs

As shown in Figure 2A, skin PAI contrast arises mainly from endogenous absorbers such as hemoglobin, melanin, collagen and lipids, whose absorption differs by wavelength. PAI does not directly visualize skin layers; instead, in post-processing, layer boundaries are recognized based on principles: melanin generally localizes to the epidermis, while the dermis contains vessels arranged from papillary capillaries to deep plexuses. However, reduced chromophore contrast or disrupted anatomical distributions, such as hypopigmentation and pigment incontinence, can interrupt this layering approach.

The etiology of most IMSDs remains incompletely understood; however, the skin’s innate and adaptive immune systems, together with epidermal keratinization, are thought to play pivotal roles in pathogenesis. In the early stages of cutaneous inflammation, vascular dilation, increased vascular permeability, and inflammatory cell recruitment occur. Immune cells and cytokines upregulate the expression of pro-angiogenic mediators (e.g., VEGF), thereby promoting vascular remodeling [36]. Consequently, alterations in vascular structure and function serve as key markers of cutaneous inflammation and are often associated with disease progression. Several IMSDs are characterized by abnormal keratinocyte proliferation and differentiation, such as psoriasis and eczema. PAI may provide information about epidermal thickness, as well as the morphology, distribution, and functional status of cutaneous vessels, which are related to pathological changes. Similarly, melanin-related signals can help map pigmentary changes during inflammatory processes.

#### 4.1.2. Advantages and Limitations of Current Applications

PAI has been shown to provide disease-relevant structural, vascular, and functional biomarkers in psoriasis, atopic dermatitis (AD), systemic sclerosis (SSc), and other immune-mediated inflammatory skin diseases (IMSDs). In patients with psoriasis and AD, PAI can detect epidermal thickening, elongation of dermal capillary loops, elevated total blood volume, and increased blood oxygen saturation. These parameters correlate with clinical scores and disease severity, and furthermore show promise for indicating subclinical disease activity and treatment response [10,11,12,17,18]. In SSc, PAI may serve as a complementary tool to nailfold capillaroscopy, displaying the various stages of nailfold capillary changes [19]. It can also probe vascular function through dynamic testing [27,28]. In psoriatic arthritis (PsA) and other IMSDs, PAI might reveal localized pathophysiological alterations or metabolic remodeling [13,14,29]. Meanwhile, raster-scanning optoacoustic mesoscopy (RSOM)-based classification models have shown preliminary ability to discriminate irritant from allergic reactions in patch testing by quantifying capillary tortuosity and dilation [30]. Collectively, the primary value of PAI in IMSDs lies in identifying early changes in lesional and perilesional tissues, providing quantitative parameters and functional indicators that suggest underlying pathophysiological or metabolic alterations.

Despite these encouraging findings, the clinical translation of PAI biomarkers remains at an early stage. First, diagnostic accuracy and validation remain incomplete. Studies are generally limited by small sample sizes, substantial heterogeneity, and cross-sectional designs. In studies assessing vascular and oxygenation parameters, factors influencing peripheral circulation—such as temperature, medications, and comorbid conditions—should be considered. We also found inconsistent conclusions across several studies [37]. The diagnostic accuracy, sensitivity, specificity, inter-device reproducibility, and optimal cut-off values of PAI in IMSDs still require determination and validation through large, multicenter studies. Histopathological validation is relatively scarce: epidermal thickness and vessel diameter measured by photoacoustic mesoscopy have been validated in some psoriasis studies, and the same biomarkers have been applied in atopic dermatitis (AD) research. In systemic sclerosis (SSc) studies, although no histopathological examination was performed, PAI quantitative parameters were compared with established imaging modalities and showed good agreement. In a reticular purpura study, vascular architecture was correlated with histopathology, but no quantitative parameter validation was conducted. Blood oxygen saturation, total blood volume and metabolic features are difficult to validate directly against histology. Models trained on PAI parameters can aid objective assessment of AD severity [16,23]; however, external validation, cross-device calibration, and interpretable feature extraction are prerequisites before any clinical deployment.

Second, subclinical observations and predictive conclusions require prospective longitudinal evidence. Differences in metabolism or structure—such as entheseal signals in PsA, vascular reactivity in SSc, or non-lesional barrier alterations in AD—are mechanistically plausible, but on their own do not establish that PAI can predict future structural damage or disease progression. Longitudinal cohort studies are needed to determine whether PAI signals can predict irreversible joint damage in PsA, fibrosis in SSc, or barrier dysfunction in AD—and whether intervention guided by PAI improves clinical outcomes. Regarding treatment monitoring, existing studies have confirmed that PAI can identify early therapeutic response or inadequate response [11,17,31], which may facilitate timely adjustment of individualized treatment. PAI may also facilitate comparative assessment of different treatment regimens [11], supporting clinical decision-making. However, these studies are not only limited by small sample sizes but also restricted to short-term changes; thus far, improvements in PAI biomarkers cannot be directly linked to sustained disease remission, reduced treatment costs, and improved quality of life. Whether patients genuinely benefit from PAI-guided management requires validation through properly designed cohort studies. Similarly, photoacoustic imaging-guided MED quantification may improve clearance rates, prevent relapse, and enhance long-term outcomes, but prospective studies are needed to confirm these benefits [38]. For topical anti-inflammatory agents, PAI can visualize drug-induced microvascular constriction in vivo and quantify drug distribution and penetration depth [34,35]; however, these experiments were conducted in mice. Future studies should verify whether such microcirculatory changes could translate to human patients and correlate with clinical outcomes—including erythema resolution, pruritus relief, flare recurrence, response duration, and corticosteroid-associated adverse effects such as skin atrophy.

#### 4.1.3. Comparison with Current Assessment Methods

Clinical scoring systems are the primary evaluation of IMSDs; however, these methods rely heavily on physician assessment of lesion appearance, introduce inherent subjectivity, and have limited capacity to detect subtle structural alterations. Imaging modalities partially address these gaps: dermatoscopy visualizes superficial dermal vasculature and epidermal scaling conveniently and is the most widely used imaging modality in dermatology. Optical coherence tomography (OCT) delineates the epidermal–dermal junction and primarily captures morphological changes in the epidermis and dermis. Reflectance confocal microscopy (RCM) provides near-cellular structural detail but is restricted to the epidermis and superficial dermis. High-frequency ultrasound (HFUS) offers the deepest penetration among these modalities, enabling precise assessment of tumor thickness, margin delineation, inflammatory activity, vascular perfusion, and filler distribution, but at comparatively lower spatial resolution [39]. In terms of vascular imaging, RSOM provides clearer and higher-contrast vascular visualization than dermoscopy, depicting the vascular tree rather than only capillaries. Dynamic OCT enables vascular imaging but suffers from axial artifacts. Ultrasound achieves vascular imaging based on the Doppler effect, yet blood flow signals are angle-dependent and it cannot visualize capillaries. RCM offers excellent resolution, but its vascular imaging is restricted to papillary dermal capillaries. We suggest that PAI should complement rather than replace current assessment modalities. Within an appropriate depth range, it noninvasively resolves signals related to hemoglobin, melanin and oxygenation, thereby adding a functional dimension that may support precision medicine. Of course, whether integrating PAI into existing clinical assessment workflows yields tangible benefits requires validation in future studies.

### 4.2. Technical Limitations Affecting Diagnostic Accuracy

In clinical applications for IMSDs, the sensitivity of photoacoustic biomarkers depends heavily on imaging accuracy. Reduced image quality may obscure subtle lesions, thereby compromising the reliability of the final assessment. The primary factors affecting image quality include spectral unmixing, spectral coloring effects and motion artifacts.

#### 4.2.1. Spectral Unmixing

Spectral unmixing requires short-pulsed excitation at variable wavelengths because multiple chromophores must be sampled across their absorption spectra. The accuracy of unmixing is limited by spectral overlap, noise, and acoustic attenuation. Moreover, many spectral unmixing methods are based on simple models; however, the light fluence distribution within human tissue is far more complex, which may introduce biases. Therefore, quantitative oxygenation or concentration estimates should be interpreted with these constraints.

#### 4.2.2. Spectral Coloring Effects

During photoacoustic imaging, light attenuation depends on tissue composition and depth. Because tissue constituents absorb and scatter different wavelengths unequally, the spectral composition of optical fluence changes with depth. This depth-dependent, wavelength-dependent fluence distortion is the basis of spectral coloring and can bias reconstructed hemoglobin, oxygenation, melanin and vascular-volume estimates. The effect is influenced by age, skin pigmentation, epidermal thickness and perfusion status [40]. Specifically, as skin pigmentation intensifies, superficial melanin absorption can reduce the fluence reaching deeper vessels, and the total vascular volume measured by PAI decreases significantly; in individuals with darker skin types (Fitzpatrick IV and V), visualizing deep dermal vascular networks becomes challenging [41].

Fernandes et al. demonstrated that increased skin melanin content is associated with elevated image noise. However, application of short-delay spatially coherent beamforming significantly improved the signal-to-noise ratio and effectively reduced imaging bias induced by skin tone differences [42].

Meanwhile, Else and colleagues used a machine-learning demixing approach based on gradient-ascent regression, in conjunction with traditional linear demixing methods, to reconstruct photoacoustic images in mouse models with varying skin melanin content. Although this approach mitigated bias in blood oxygen measurements, overestimation of blood oxygen concentration persisted in pigmented skin models [43]. These data show that current correction methods can reduce but not eliminate skin-tone-related bias, particularly when superficial melanin absorption dominates local fluence. Consequently, vascular and oxygenation metrics in highly pigmented tissues should be interpreted cautiously and validated with representative skin phototypes.

#### 4.2.3. Motion Artifacts

Currently, the majority of PAI systems rely on mechanical scanning approaches. Because scanning duration is relatively long, motion artifacts are prone to occur during image reconstruction. This issue is particularly prominent in body regions significantly affected by respiratory motion, such as the chest and abdomen. To address this challenge, researchers commonly utilize motion correction algorithms during image reconstruction; however, these algorithms still fail to fully offset the adverse effects of tissue displacement on image quality.

In clinical settings, motion artifacts may reduce sensitivity to subtle lesions, create false biomarker shifts and increase diagnostic misclassification. Motion is particularly relevant for long mechanical scans, curved surfaces, pediatric participants and sites affected by respiration or involuntary movement. Standardized positioning, acquisition speed, motion correction and quality-control criteria are therefore essential for reproducible diagnostic use.

Recent studies have shown that increasing scanning speed can effectively mitigate this problem. For instance, He et al. [44] improved imaging speed by instructing patients to hold their breath during data acquisition, thereby reducing motion artifacts while preserving image quality. Similarly, Huynh et al. [45] developed a novel 3D photoacoustic scanner based on Fabry-Pérot sensors. By parallelizing sensor readings, increasing the laser pulse repetition rate, and integrating compressed sensing, this device reduced scanning time from several minutes to seconds or even hundreds of milliseconds, markedly improving scanning speed while maintaining high spatial resolution.

Although these newly developed high-speed imaging devices can effectively alleviate motion-related artifacts, widespread adoption across regions and imaging platforms remains an unresolved challenge.

### 4.3. Technical Challenges and Economic Costs

In summary, changes in cutaneous capillaries are a key hallmark of IMSDs; therefore, high-resolution, accurate imaging equipment is required for their evaluation. The acquisition of high-quality images depends on high-performance optical and acoustic components, advanced image processing technologies, and careful balancing of laser intensity with biosafety requirements. These technical challenges have largely limited current PAI equipment to laboratory settings, hindering its introduction and widespread adoption in clinical institutions.

On the one hand, this cutting-edge technology is difficult to share across institutions and geographic regions; on the other hand, its substantial cost constitutes a major barrier to adoption by medical institutions. By enabling point-of-care assessment, affordable portable PAI lowers the threshold for large multicenter studies, generating real-world evidence that may ultimately facilitate clinical adoption. There are various strategies, such as replacing traditional lasers with more cost-effective LED light sources, integrating PAI modules into existing medical ultrasound devices and employing Fabry–Pérot interferometers (FPIs), whose sensitivity does not degrade with decreasing element size. However, whether these optimization measures can reduce costs while maintaining image quality and clinical utility remains to be validated with additional empirical data.

### 4.4. Strengths and Limitations of the Study

This review has several strengths. It focuses specifically on IMSDs rather than dermatologic PAI in general, integrates diagnostic and therapeutic applications, distinguishes major PAI modalities, and highlights disease-specific biomarkers together with translational barriers. The review also incorporates recent work on skin phototype bias, high-speed scanning and clinical translation roadmaps.

The limitations should also be acknowledged. As a narrative review, this article does not provide pooled diagnostic accuracy estimates or formal meta-analysis. The underlying evidence base is dominated by small, heterogeneous, single-center feasibility studies and prototype imaging systems. Study populations often have limited representation of darker skin phototypes, external validation is scarce, acquisition protocols and biomarker definitions differ across teams, and positive publication bias is possible. These factors limit generalizability and prevent direct clinical adoption of most PAI biomarkers at present.

### 4.5. Future Directions

Researchers and industry stakeholders have recognized the current challenges in the clinical translation of PAI. In 2023, the International Photoacoustic Standardization Alliance (IPSA) announced a roadmap for the clinical translation of PAI, covering four key areas: data management, standardization, testing models and methods, and clinical applications. This roadmap aims to promote the standardization and commercialization of photoacoustic devices [8].

Existing findings are promising, but they primarily demonstrate the feasibility of PAI for clinical applications in IMSDs. Translating PAI into routine clinical practice requires several key initiatives: establishing standardized protocols, conducting large-scale prospective multicenter validation studies, optimizing hardware and technology, and reducing costs.

Standardized protocols should be developed for different IMSDs and clinical scenarios. These protocols need to specify optimal PAI configurations for specific diseases and anatomical sites, standardize biomarker extraction methods, and define diagnostic thresholds, while also addressing practical factors such as patient positioning and temperature control. Further studies are needed to verify the reproducibility of PAI-derived metrics and their correlations with histopathological and molecular features. Large multicenter prospective trials should be designed to compare PAI with clinical scores and established skin imaging modalities, evaluating whether it can streamline clinical workflows, improve long-term outcomes, and deliver clinical and economic benefits.

Technical development should aim for portable, cost-effective systems, faster scanning speeds, reproducible calibration, robust skin-tone correction, open benchmark datasets, and interoperable software pipelines. This necessitates a collaborative framework bringing together dermatologists, radiologists, biomedical engineers, medical IT specialists, and industry partners, to ensure that technological breakthroughs genuinely meet clinical needs.

## 5. Conclusions

As an emerging biomedical imaging technique, PAI enables high-resolution functional imaging of skin, providing simultaneous assessment of microvascular architecture, hemoglobin oxygenation, and melanin distribution. It can detect physiological and pathological changes at inflammatory sites and remains a promising complementary tool for the diagnosis and management of IMSDs. Future interdisciplinary collaboration should focus on standardized protocols, multicenter prospective validation, and improved device performance before routine clinical translation.

## Figures and Tables

**Table 1:** Summary of key included studies evaluating photoacoustic imaging in immune-mediated inflammatory skin diseases.

Application	Study	Sample Size/Design	PAI Modality	Comparator	Main Findings	Limitations
Psoriasis diagnosis	Aguirre et al. 2017 [10]	Exploratory clinical study; 6 psoriasis patients	Ultra-broadband RSOM	Clinical assessment; histopathology for selected structures	Epidermal thickness, dermal microvascular parameters, and OPIND reflected psoriasis morphology and severity.	Small cohort; limited external validation; thresholds not standardized.
Psoriasis treatment monitoring	Hindelang et al. 2022 [11]	Clinical monitoring; 80 measurements from 20 plaques	RSOM	PASI and treatment response	PAI indices decreased after treatment and captured vascular changes before visible clinical improvement.	Single-center/prototype workflow; sensitivity and specificity across therapies remain unclear.
Psoriasis severity and response	Li et al. 2024 [12]	Clinical cohort; 8 psoriasis patients	High-resolution multispectral optoacoustic imaging	Clinical severity and lesion status	Oxygenation and vascular volume were associated with psoriasis severity and non-lesional changes.	Histopathological validation and diverse skin phototype data remain limited.
Psoriatic arthritis	Hallasch et al. 2021 [13]	Exploratory clinical study; 22 psoriasis/PsA-related patients and 19 healthy volunteers	Multispectral optoacoustic tomography (MSOT)	Clinical PsA evaluation; conventional radiography/HFUS	Enthesis oxygenation and hemoglobin signals suggested metabolic alterations before structural damage.	Predictive value requires larger longitudinal studies.
Psoriasis/PsA entheses	Fagni et al. 2024 [14]	Cross-sectional MAPSA study; 30 psoriasis, 30 PsA, and 30 healthy controls	MSOT	Clinical group comparison	Oxygenated hemoglobin and oxygen saturation increased at entheses, supporting metabolic remodeling.	Cross-sectional design cannot establish progression or prediction.
Atopic dermatitis biomarkers	Li et al. 2022 [15]	Clinical study; 16 AD patients	Multispectral RSOM	Lesional, non-lesional and control skin	Structural and vascular markers differentiated lesional from non-lesional AD skin.	Small cohort; histological or molecular confirmation is still needed.
Atopic dermatitis severity	Park et al. 2021 [16]	Model-learning study; 53 AD patients and 23 healthy controls	3D optoacoustic mesoscopy	Clinical severity labels	Machine-learning models classified AD severity from PAI-derived 3D features.	Model requires external validation and prospective testing.
Atopic dermatitis treatment	Yew et al. 2019 [17]	Single-patient longitudinal observation	RSOM with confocal Raman spectroscopy	Dupilumab treatment course	PAI showed vascular and morphological changes during treatment.	Case-level evidence; no generalizable diagnostic accuracy estimate.
Systemic sclerosis nailfold	Nitkunanantharajah et al. 2020 [18]	Clinical cohort; 23 SSc and 19 controls	3D optoacoustic imaging	Nailfold capillaroscopy/clinical diagnosis	Nailfold vascular morphology supported disease differentiation.	Cut-off values and multicenter reproducibility are not established.
Systemic sclerosis skin/finger assessment	Daoudi et al. 2021 [19]	Clinical cohort; 17 SSc, 5 primary Raynaud’s, and 9 healthy controls	PAI with HFUS	HFUS and clinical groups	Lower oxygen saturation and altered skin thickness were observed in SSc.	Measurements may be affected by temperature, pressure, skin site, and device settings.

**Table 2:** Comparison of conventional cutaneous imaging modalities and photoacoustic imaging for skin assessment.

Imaging Modality	Maximum Penetration Depth	Spatial Resolution	Functional Detection Capability	Main Advantages	Main Limitations
Dermatoscopy	Epidermis and superficial dermis	Medium	Poor	Low cost, easy operation, suitable for pigmentary lesions	Shallow penetration, no quantitative functional indicators
Optical Coherence Tomography (OCT)	~1.0 mm	High	Limited blood flow mapping	Clear dermal–epidermal junction imaging	Restricted to superficial skin layers, axial artifacts
Reflectance Confocal Microscopy (RCM)	~0.3 mm	Ultra-high (subcellular)	Poor	Consistent with histopathology; high microstructural resolution	Extremely shallow penetration, limited to epidermis and dermis papillary layer
Cutaneous Ultrasound	Full-thickness skin	Low	Basic blood flow evaluation	Excellent deep tissue penetration, low cost	Low spatial resolution, no molecular/functional imaging
Photoacoustic Imaging (PAI)	2–5 mm	High	Strong (hemoglobin, melanin, oxygen saturation, collagen)	Combines deep penetration with high resolution; multi-component functional quantification	Spectral coloring artifacts, motion artifacts, high equipment cost, and no unified standards

Note: PAI performance is modality-dependent; penetration, resolution, field of view and quantification accuracy vary substantially among PAT, PAM, PAMes and RSOM systems.

## Data Availability

No new data were created or analyzed in this study. Data sharing is not applicable to this article.

## Abbreviations

The following abbreviations are used in this manuscript:

AD	Atopic Dermatitis
EASI	Eczema Area and Severity Index
IMSDs	Immune-Mediated Inflammatory Skin Diseases
MED	Minimal Erythema Dose
OCT	Optical Coherence Tomography
OPIND	Optoacoustic Index
PAI	Photoacoustic Imaging
PASI	Psoriasis Area and Severity Index
RCM	Reflectance Confocal Microscopy
RSOM	Raster-Scan Optoacoustic Mesoscopy
SCORAD	Scoring of Atopic Dermatitis
SSc	Systemic Sclerosis
UV	Ultraviolet
